# Hepatitis C Virus: Epidemiological Challenges and Global Strategies for Elimination

**DOI:** 10.3390/v17081069

**Published:** 2025-07-31

**Authors:** Daniela Toma, Lucreția Anghel, Diana Patraș, Anamaria Ciubară

**Affiliations:** 1 Doctoral School of Biomedical Sciences, “Dunarea de Jos” University Galati, 800008 Galati, Romania; 2 Saint Apostle Andrew Emergency County Clinical Hospital, 177 Brailei St., 800578 Galati, Romania; 3 Faculty of Medicine and Pharmacy, “Dunarea de Jos” University of Galati, 35 AI Cuza St., 800010 Galati, Romania

**Keywords:** hepatitis C virus, epidemiology, modes of transmission, infection management, early detection, vulnerable populations

## Abstract

The global elimination of hepatitis C virus (HCV) has been prioritized by the World Health Organization (WHO) as a key public health target, with a deadline set for 2030. This initiative aims to significantly reduce both new infection rates and HCV-associated mortality. A major breakthrough in achieving this goal has been the development of direct-acting antiviral agents (DAAs), which offer cure rates exceeding 95%, along with excellent safety and tolerability. Nevertheless, transmission via parenteral routes continues to be the dominant pathway, particularly among high-risk groups, such as individuals who inject drugs, incarcerated populations, those exposed to unsafe medical practices, and healthcare professionals. Identifying, monitoring, and delivering tailored interventions to these groups is crucial to interrupt ongoing transmission and to reduce the burden of chronic liver disease. On a global scale, several nations have demonstrated measurable progress toward HCV elimination, with some nearing the targets set by WHO. These achievements have largely resulted from context-adapted policies that enhanced diagnostic and therapeutic access while emphasizing outreach to vulnerable communities. This review synthesizes current advancements in HCV prevention and control and proposes strategic frameworks to expedite global elimination efforts.

## 1. Introduction

HCV infection continues to represent a critical global health issue, despite significant advances in antiviral treatment and diagnostic methods over the past decade. As a blood-transmitted pathogen, HCV plays a critical role in the development of chronic liver diseases, notably cirrhosis and hepatocellular carcinoma (HCC), remaining a key contributor to liver-related morbidity and mortality on a global scale. According to recent estimates from the WHO, approximately one million new HCV infections occur annually, reflecting the ongoing challenges in controlling viral transmission at a global level [1,2]. In 2022, hepatitis C was associated with nearly 242,000 deaths, underscoring the severe consequences of delayed diagnosis and limited access to effective therapies [1].

These figures highlight an urgent need to reassess global strategies for prevention and control. The global distribution of HCV infection reveals considerable regional differences. Prevalence is highest in low- and middle-income countries (LMICs), where healthcare systems often struggle to provide comprehensive screening, timely diagnosis, and access to antiviral therapy. Importantly, nearly four out of five individuals living with chronic HCV remain unaware of their infection, limiting their opportunity to benefit from curative treatments. Even among those diagnosed, only about 5% initiate therapy, highlighting persistent gaps between diagnosis and treatment [2].

Historically, the standard of care involved PEGylated interferon alpha (PEG-IFNα) combined with ribavirin (RBV)—a regimen with moderate efficacy, long treatment duration, and frequent adverse effects, which negatively impacted adherence and outcomes [3]. The year 2014 marked a pivotal advancement in the therapeutic management of hepatitis C, with the introduction of DAAs revolutionizing treatment efficacy and clinical outcomes. These highly effective, well-tolerated oral regimens have transformed HCV management, allowing viral clearance in most patients within 8 to 12 weeks of therapy. Consequently, hepatitis C has evolved from a chronic, difficult-to-treat infection into one with curative potential for the majority of cases [4].

Today, two pangenotypic DAA combinations—sofosbuvir/velpatasvir and glecaprevir/pibrentasvir—are widely used as first-line treatments. Their proven efficacy across all HCV genotypes and favorable safety profiles make them essential tools in national and international elimination strategies. These regimens support large-scale treatment initiatives, even among patients with comorbidities or advanced liver disease [5,6,7].

In light of these advances, the global health community has intensified elimination efforts. During its sixty-ninth session in 2016, the World Health Assembly approved a resolution targeting the elimination of viral hepatitis as a pressing global public health issue by 2030. The WHO strategy set clear targets: a 65% reduction in hepatitis-related mortality, an 80% reduction in new infections, diagnosis of at least 90% of infected individuals, and treatment of at least 80% of those diagnosed [5]. To facilitate national monitoring, the WHO also established quantitative elimination benchmarks, including no more than five new HCV infections per 100,000 individuals annually and no more than two hepatitis C-related deaths per 100,000 people per year [8].

Although there has been important progress, several ongoing challenges still stand in the way of fully reaching the goals for eliminating hepatitis C. Among the most significant is the risk of HCV reinfection following successful antiviral therapy. Reinfection is suspected when viremia reappears after initial viral clearance, confirmed through sensitive molecular testing. Achieving an undetectable level of HCV RNA (hepatitis C virus ribonucleic acid) 12 weeks after completing therapy—commonly referred to as sustained virologic response (SVR12)—continues to serve as the primary indicator of successful hepatitis C treatment outcomes. However, recurrence of viremia may result from either relapse of the initial infection or a new exposure, particularly in high-risk populations [9].

Achieving sustained virologic response (SVR) is strongly associated with favorable clinical outcomes, including normalization of liver enzyme levels, fibrosis regression, and improved hepatic function. Nevertheless, viral eradication does not fully eliminate the risk of hepatocellular carcinoma or liver-related mortality, especially among patients with pre-existing cirrhosis or coexistent liver disease. Additional factors, such as metabolic syndrome, harmful alcohol use, and hepatitis B virus (HBV) co-infection, may further impact long-term liver health even after HCV clearance [10].

In recent decades, HCV epidemiology has evolved considerably, with marked shifts in its global distribution and transmission dynamics, shaped by demographic transitions, public health interventions, and changes in risk behaviors. Historically, unscreened blood transfusions and unsafe medical practices were the main transmission routes. Thanks to rigorous safety measures, these pathways have been largely controlled in high-income countries. In contrast, in many low-resource settings, these risks persist, reflecting broader healthcare disparities [11].

Currently, certain key populations remain central to the epidemic. People who inject drugs (PWIDs) and Human Immunodeficiency Virus (HIV)-positive men who have sex with men (MSMs) experience the highest rates of ongoing HCV transmission. These groups act as critical reservoirs for the virus, even where healthcare-associated transmission has been largely curtailed. Therefore, elimination strategies must include targeted interventions for these vulnerable groups, focusing on prevention, testing, and treatment programs. Additionally, vertical transmission remains the main source of HCV infection among children, highlighting the importance of maternal screening and appropriate management during pregnancy [12].

The incarcerated population represents a globally important high-risk group in the HCV epidemic. According to a recent meta-analysis, the global prevalence of HCV among people in prison is estimated at 17.7%, with regional variations reaching 28.4% in Oceania and 25.1% in Europe [13]. This elevated burden is primarily driven by overlapping risk factors such as injection drug use (IDU), unsafe tattooing, and limited access to healthcare services both prior to and during incarceration [14]. Despite the high risk, HCV testing and treatment coverage remain suboptimal in many correctional settings [15]. However, successful national models—such as those in Australia and Spain—demonstrate that prison-based programs incorporating universal screening and timely initiation of DAA therapy can achieve high cure rates and reduce post-release transmission [16,17]. The WHO emphasizes the inclusion of prison-based health services in national elimination plans, advocating for universal testing and linkage to care within carceral systems as a critical step toward achieving the 2030 elimination targets [15,18].

Although current strategies for eliminating HCV infection primarily focus on key populations, including PWIDs, MSMs, and prisoners, international guidelines and the recent literature emphasize the need to expand screening to the general population in settings where prevalence is significant. This broader approach can be implemented either through universal screening or by targeting specific birth cohorts, depending on the local epidemiological context. According to the European Centre for Disease Prevention and Control (ECDC) guidelines, population-based screening is recommended in countries with high prevalence, particularly among individuals born between 1945 and 1965 who are considered to have been exposed to iatrogenic risks in previous decades [19]. Furthermore, the WHO underscores that general population testing can play a decisive role in achieving the 2030 global elimination goals when adapted to the national context [20].

Building on these global recommendations, this review provides a comparative analysis of national and regional strategies for HCV elimination, with a particular focus on high-performing programs in Egypt, Spain, and Australia. By evaluating the determinants of their success and the barriers encountered in different epidemiological and socioeconomic contexts, this study seeks to highlight existing knowledge gaps and outline future directions required to optimize screening, diagnosis, and treatment strategies. The ultimate goal is to inform evidence-based policies that can accelerate global progress toward achieving the WHO 2030 hepatitis C elimination targets.

## 2. Methodology

This structured literature review aimed to synthesize current evidence on global efforts to prevent, control, and eliminate HCV. Relevant, up-to-date research was identified through a comprehensive search of these major international databases—PubMed, Scopus, Web of Science—which are widely recognized for their extensive coverage of biomedical and public health research.

This research targeted English-language, open-access publications available in full-text format, published between 2010 and May 2025, including online-ahead-of-print papers. This timeframe ensured the inclusion of recent developments in HCV screening, elimination strategies, and public health interventions. Search terms included combinations such as “hepatitis C elimination”, “direct-acting antivirals”, “HCV screening”, “vulnerable populations”, and “global HCV control”, along with additional terms, including “HCV epidemiology”, “prevention”, and “risk factors”. The application of Boolean operators such as AND and OR was employed to refine the search strategy, ensuring an optimal balance between sensitivity and specificity.

An initial assessment of titles and abstracts was conducted to remove duplicate records, non-English language sources, and studies considered irrelevant to the objectives of the review. The inclusion criteria were limited to studies published within the past 15 years that provided unrestricted access to the full text. Eligible study types included original research, observational studies, intervention studies, systematic reviews, meta-analyses, clinical guidelines, and case studies addressing HCV elimination and related strategies.

Articles were excluded if they were older than 15 years, written in languages other than English, or not available in full text or focused on other types of viral hepatitis.

**Figure 1:**
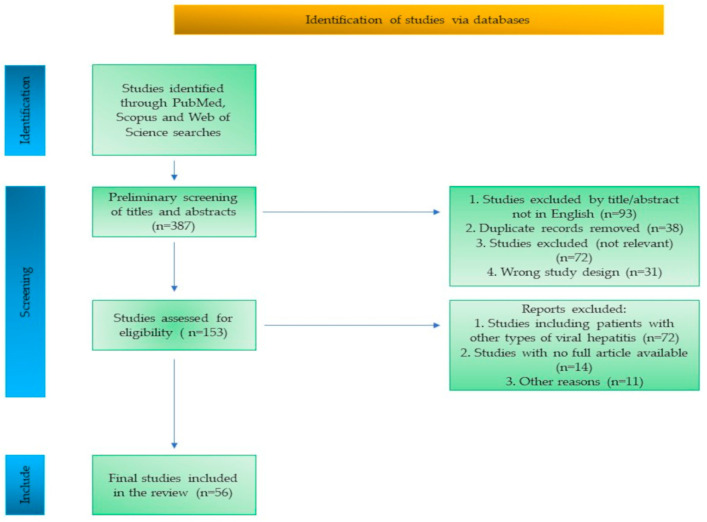
Flow diagram of the study selection process.

The study selection process is summarized in a flow diagram (Figure 1), outlining the identification, screening, eligibility assessment, and inclusion of studies.

Full-text versions of selected studies were retrieved and systematically analyzed. Data extraction focused on elimination strategies, intervention for high-risk populations, treatment approaches, implementation models, and progress toward WHO targets. The content of this manuscript is derived from a comprehensive synthesis of the reviewed literature.

## 3. History

Efforts to uncover the viral causes of hepatitis began in the mid-20th century, representing a significant development in the field of hepatology. In 1965, Blumberg and his team identified a blood antigen later named the hepatitis B surface antigen (HBsAg), thereby confirming HBV as a major causative agent of serum hepatitis. This discovery led to the development of diagnostic assays for HBsAg detection and their widespread use in blood transfusion screening, reducing the incidence of post-transfusion hepatitis by nearly 50%. However, hepatitis cases still occurred despite HBsAg-negative blood donations, suggesting the presence of other unidentified agents [21].

In 1975, Alter and collaborators introduced the term “non-A, non-B hepatitis” to classify cases of viral liver inflammation that were not linked to hepatitis A or B viruses. Their research involved transfusing blood from chronically infected donors into chimpanzees, which subsequently developed liver inflammation and elevated alanine aminotransferase levels. These findings provided strong experimental evidence for a new infectious agent [22].

The identity of this agent remained unknown until 1989, when Houghton and his team successfully isolated and sequenced its genome, officially identifying it as HCV. The identification of HCV represented a critical milestone that facilitated the development of reliable diagnostic tools and the implementation of stringent blood donor screening practices, leading to a substantial decrease in transfusion-related infections [21].

In 2020, international recognition of the discovery’s impact came when Harvey J. Alter, Michael Houghton, and Charles M. Rice were honored with the Nobel Prize in Physiology or Medicine, highlighting their distinct but complementary roles in uncovering the virus responsible for previously unclassified cases of transfusion-related hepatitis. Through meticulous clinical studies, Harvey J. Alter demonstrated that certain post-transfusion hepatitis cases could not be explained by known hepatitis viruses, strongly suggesting the existence of an unidentified infectious cause. Houghton subsequently achieved its genetic isolation, while Rice validated the virus’s pathogenic potential using experimental model systems. Together, their contributions elucidated the cause of what had long been classified as non-A, non-B hepatitis, establishing a basis for advancements in its diagnosis, prevention, and therapy. The recognition of HCV as a distinct RNA virus reshaped the management of chronic viral hepatitis and continues to influence global public health strategies [2,23,24].

## 4. Characteristics of the HCV Genome

**Figure 2:**
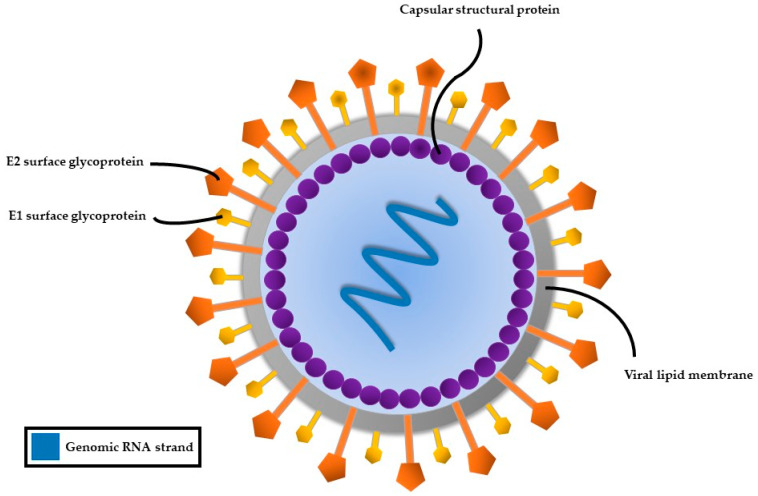
Structure of the hepatitis C virus.

**Figure 3:**
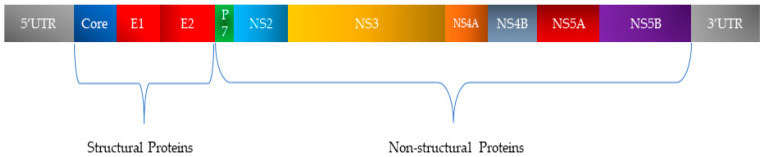
Organization of HCV genetic material and classification of encoded proteins.

HCV, a member of the Flaviviridae family, possesses a single-stranded RNA genome enclosed in a small, enveloped virion approximately 50 nanometers in diameter (Figure 2). The RNA genome of HCV features one continuous coding region, which is translated into a precursor polyprotein of nearly 3100 amino acids. This polyprotein is cleaved by both host and viral proteases into individual structural and non-structural proteins required for viral replication and assembly (Figure 3). The principal structural components of the virus comprise the core protein, responsible for nucleocapsid formation, along with two envelope glycoproteins, E1 and E2, which are anchored in the surrounding lipid bilayer. These envelope proteins are essential for mediating the virus’s attachment to and penetration of host cells, with E2 protein playing an additional role in evading host immune responses [25].

In addition, HCV encodes seven non-structural proteins (p7, NS2, NS3, NS4A, NS4B, NS5A, and NS5B) that coordinate RNA replication, virion assembly, and release from infected cells [25,26].

A key genomic feature is the presence of two hypervariable regions within the E2 protein. HCV exhibits a high rate of genetic variation, primarily due to its fast replication process and the lack of error-correcting ability in its RNA-dependent RNA polymerase. This results in significant genetic variability, enabling rapid adaptation and evasion of host immune responses [25,27].

After initial infection, HCV RNA can be identified in the bloodstream as early as one to four weeks post-exposure, with viral titers generally reaching their peak between the eighth and twelfth week. Although some individuals spontaneously clear the infection, 50–85% develop chronic hepatitis C, often due to insufficient CD4+ T-lymphocyte and CD8+ cytotoxic T-lymphocyte responses. HCV does not directly induce hepatocyte death. Instead, liver injury arises from persistent immune-mediated inflammation. Chronic immune activation promotes hepatic fibrosis, which can progress to cirrhosis over time. Disease progression is influenced by factors such as excessive alcohol intake, co-infection with HIV or HBV, HCV genotype 3 infection, obesity, insulin resistance, and non-alcoholic fatty liver disease (NAFLD). These molecular and biological features contribute to HCV’s persistence and help explain why chronic infection frequently leads to severe liver complications [28].

## 5. The Worldwide Prevalence of Hepatitis C Virus Genotypes

HCV exhibits remarkable genetic diversity, which has led to its classification into eight genotypes and at least 93 subtypes, with a minimum nucleotide sequence divergence of 30% between genotypes. This genetic heterogeneity not only defines viral classification but also influences epidemiological patterns, clinical progression, and therapeutic responses [29].

**Figure 4:**
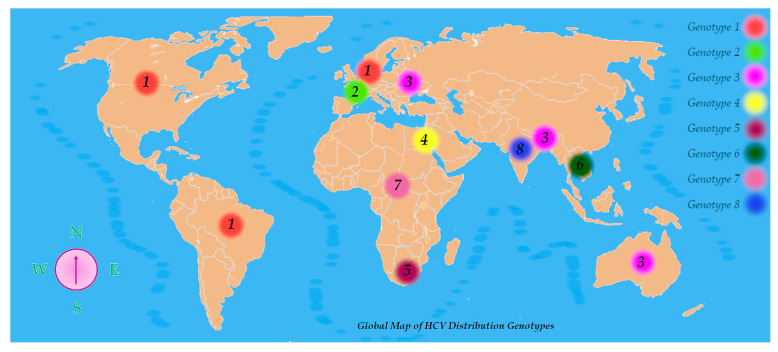
Global map of HCV distribution genotypes.

HCV genotypes display distinct global distributions. Genotypes 1, 2, and 3 are the most prevalent HCV variations found worldwide among those that have been characterized. Genotype 1 is most frequently encountered in regions such as North America, Northern and Western Europe, and areas of South America. Genotype 2 is also common in Western nations. Genotype 3 prevails in South Asia, Australia, and certain parts of Europe. Genotype 4 shows a high prevalence across the Middle East and North Africa, with notably elevated rates in Egypt and Saudi Arabia. Genotype 5 has been reported mainly in Southern Africa, genotype 6 in Southeast Asia, and genotype 7 has been detected primarily in Central African populations. The recent identification of genotype 8 in India further underscores HCV’s genetic complexity (Figure 4) [29].

This variability has significant clinical implications. Among the various HCV genotypes, type 3 is consistently correlated with a more aggressive clinical course of liver disease. Patients infected with genotype 3 have a higher rate of fibrosis progression and earlier onset of cirrhosis compared to those with other genotypes [26,30].

Prior to the development of DAAs, interferon- and ribavirin-based therapies showed variable efficacy by genotype: genotypes 1a and 1b had lower response rates, whereas 3a and 3b responded more favorably. Although DAA regimens have significantly improved overall cure rates and safety profiles, genotype 3 still presents challenges, particularly in treatment response and resistance patterns [29,30].

Consequently, accurate genotyping remains a critical component in clinical management of HCV, guiding the selection of antiviral regimens and treatment duration. A comprehensive understanding of the global distribution and biological behavior of HCV genotypes is essential for optimizing patient care and informing hepatitis C elimination strategies worldwide.

## 6. Modes of Transmission of HCV

The principal route of HCV transmission involves direct contact with blood contaminated by the virus. Historically, the main route of transmission was through blood transfusions and the use of unscreened blood products. With the implementation of advanced serological and molecular screening methods, the risk associated with transfusions has been significantly reduced. IDU remains the primary pathway for HCV transmission today, with the sharing of contaminated needles and injection equipment posing the highest risk among PWIDs [2,31].

To provide a more comprehensive understanding of the epidemiological dynamics of HCV transmission, it is essential to examine data derived from original research conducted in various global contexts. These studies offer granular insights into how specific transmission routes operate within distinct population groups and settings, revealing the interplay between medical practices, behavioral risks, and social determinants. Table 1 presents a selection of original research articles, each focused on a particular mode of HCV transmission, along with details regarding the study population, geographic region, year of publication, and main methodological limitations (sample size, design constraints, and potential sources of bias). This synthesis supports the formulation of evidence-based, context-sensitive public health strategies aimed at interrupting transmission chains and advancing toward HCV elimination.

Among the additional and context-specific transmission routes highlighted in the literature are unsafe medical or dental procedures involving unsterilized instruments, as well as cosmetic practices such as tattooing, body piercing, and acupuncture when performed without proper hygiene standards. Healthcare workers are also at risk of accidental exposure via needlestick injuries or contact with sharp instruments used on infected patients.

Each year, an estimated 112 million blood donations are collected worldwide, highlighting the importance of robust screening procedures to ensure transfusion safety. WHO recommends that all blood intended for transfusion be systematically screened for infections such as HIV, HBV, HCV, and syphilis to ensure safety [39].

IDU constitutes a leading factor in the global spread of HCV, with PWIDs being disproportionately affected. Genotypes 1a and 3a are the most frequently detected in this population. Subtype 3a, originally endemic in Southeast Asia, has spread among drug users in Europe and North America. Data from several European countries reveal an increasing circulation of HCV genotypes 1a and 3a, particularly in Germany, France, Italy, and Portugal. Instances of dual HCV genotype infections have been reported across Europe, including combinations like 1b and 3a in Italy; 2a and 3b in Germany; and 1a, alongside 1b, in Sweden. The rapid expansion of HCV in Eastern Europe and Central Asia is linked to heroin injection and the emergence of synthetic drugs. Genotype 3a has shown a rising prevalence in countries such as Romania, Bulgaria, and Poland, and across the Balkan region [40,41].

PWIDs play a significant role in shaping the transmission patterns of HCV, influencing not only high-risk groups but also broader population networks [42]. This dynamic is exacerbated by factors such as poverty, incarceration, and co-infection with HIV. In rural parts of the United States, PWIDs are at increased risk due to limited healthcare infrastructure, lack of harm reduction programs, and reduced access to opioid use disorder treatments. Rural populations experience increased HCV prevalence as a consequence of these inequities, when compared to urban settings [43].

MSMs represent another high-risk group, with multiple HCV outbreaks reported across Europe, North America, Australia, and Asia. In regions where homosexuality is stigmatized, such as parts of the Arabian Peninsula, barriers to care and social discrimination further increase vulnerability. HIV and HCV co-infections are frequently reported in PWIDs and MSMs, reflecting shared transmission risks in these key populations. In some regions, up to 25% of individuals living with HIV are also infected with HCV. Among MSMs living with HIV, the rate of HCV co-infection is notably elevated, reflecting the overlapping modes of transmission [40].

Although sexual transmission of HCV is best documented among MSMs, recent evidence indicates that this route is not exclusive to this population. Heterosexual transmission, while less efficient than for other viruses, such as HIV, is not negligible under certain circumstances, including unprotected sexual contact, the presence of genital lesions, drug use during sexual activity, or co-infection with other sexually transmitted infections [38,44,45]. Furthermore, the 2020 ECDC report documented sexual transmission in heterosexual populations engaging in high-risk behaviors, confirming the need for a broader preventive approach [46].

HCV prevalence is highly concentrated geographically. Around 30 countries account for 80% of global cases, with the highest burdens observed in China, India, Pakistan, Ukraine, Russia, and the United States of America (USA). Over 70% of people with active HCV infection reside in LMICs, where access to testing and treatment remains limited. For example, infection rates in Ukraine and Romania reach 3.1% and 2.3%, respectively, compared to less than 1% in most Western European nations [11].

The highest numbers of PWIDs are reported in countries like China, Russia, the USA, and Brazil, with significant populations also found in Mexico, Pakistan, and Thailand. In nearly all European countries, HCV seroprevalence among PWIDs is high, with particularly elevated rates in Latvia, Portugal, Turkey, and Cyprus. In contrast, lower prevalence (<30%) is reported in the Czech Republic, Hungary, and Slovenia. A favorable decline in HCV transmission has been observed in Germany, France, the United Kingdom (UK), and Italy in recent years [40].

In LMICs, unsafe medical practices—including reuse of unsterilized syringes and inadequately screened blood transfusions—remain significant contributors to HCV transmission. Among hemodialysis patients, infection rates can exceed 40% in some areas of the Arabian Peninsula and in China. Co-infection with HBV is also reported, with HCV co-infection rates ranging from 3% in Thailand to 30% in Spain [40].

Vertical transmission of HCV is more likely when the mother has detectable levels of viral RNA, and this risk increases significantly if she is also infected with HIV. Antiretroviral therapy for HIV during pregnancy reduces the risk, aligning transmission rates with those seen in mothers with HCV monoinfection [47].

A nuanced understanding of transmission dynamics is essential for effective prevention strategies and global elimination efforts.

## 7. Prognosis

The clinical course of HCV infection is marked by significant variability, influenced by viral persistence and host-specific factors. Although spontaneous clearance can occur, it is observed in only 10–15% of cases. In the remaining majority, the infection persists and may progress to chronic liver disease [28]. About one in five people with chronic HCV develops cirrhosis within two decades [48]. In this group, HCC risk over three decades is 1–5%. Progression may accelerate in older adults or those with metabolic comorbidities. Liver damage risk increases with high alcohol intake. Pre-existing cirrhosis or HBV co-infection further amplifies this risk and leads to poorer outcomes [28,49].

Viral eradication, marked by undetectable HCV RNA post-treatment, is key to long-term success. Achieving SVR significantly reduces cirrhosis, HCC, and liver-related mortality. Patients with SVR have better survival and slower liver function decline, regardless of disease stage [28,50].

## 8. Screening, Prevention, and Control Strategies

Timely diagnosis represents a fundamental component in the effective management of HCV, serving as the initial step within the comprehensive care pathway. When followed by prompt linkage to treatment, it facilitates access to curative therapy and helps reduce viral transmission. In recognition of its critical role, the WHO recommends universal HCV testing within correctional facilities, where prevalence rates are significantly elevated [51].

Diagnosis typically begins with antibody screening for HCV, followed by RNA-based testing to confirm ongoing viral activity. This dual-step strategy ensures both diagnostic precision and informed clinical decision-making [5].

One of the main obstacles in HCV control is the high proportion of asymptomatic individuals who remain undiagnosed [52]. Broadening access to testing is essential—not only for case identification but also to reduce community-level spread. Reflecting this need, professional associations such as the American Association for the Study of Liver Diseases advocate routine HCV screening for all adults aged 18 and older [53].

Advancements in diagnostic approaches continue to enhance detection accuracy. For example, recent findings have shown that pairing two complementary screening methods significantly improves sensitivity and specificity, surpassing the reliability of single-test protocols. This supports adopting dual-testing strategies, especially in high-burden or resource-limited settings [39,54,55].

Approaches to HCV screening vary significantly across countries, influenced by healthcare system capacity, policy frameworks and population risk profiles. Table 2 presents a comparative overview of national HCV screening strategies and the diagnostic methods currently implemented.

Rapid tests used at the point of care offer a viable option, especially where conventional lab diagnostics are not accessible. Particularly in remote or underserved regions, these devices enable faster diagnosis and improved outreach to hard-to-reach populations.

An increasingly recognized component of strategies aimed to eliminate blood-borne viral (BBV) infections in the implementation of screening programs in emergency departments. Evidence shows that integrating HCV testing into emergency care can significantly increase early diagnosis rates in densely populated urban settings [57]. Systematic BBV screening in emergency departments in Spain led to a substantial increase in diagnosis and referral rates for treatment [67], while studies conducted in South Korea indicate that this approach is sustainable, cost effective, and well accepted by patients, particularly when supported by educational interventions and integrated health information systems [68].

Preventive interventions are equally vital in curbing new HCV infections. Key measures include rigorous infection control practices in clinical settings—such as safe injection techniques, appropriate disposal of medical waste, and thorough sterilization of equipment. For PWIDs, essential harm-reduction services—like needle exchange programs, OAT, and counseling—are proven to reduce transmission. Continued mandatory screening of donated blood, alongside public education on safe sexual behaviors, reinforces a comprehensive prevention framework [1].

Some countries, such as Italy, have adopted integrated public health policies that provide free HCV and HIV screening and treatment. These programs are often jointly coordinated by health and justice ministries, though their implementation may vary regionally depending on local healthcare infrastructure [69].

In high-income nations, routine screening at blood donation centers typically involves antibody testing using enzyme-linked immunosorbent assay (ELISA) or chemiluminescence immunoassay (CLIA), followed by nucleic acid amplification testing (NAT) for RNA confirmation. Since their broad implementation in the 1990s, these testing protocols have almost entirely removed the threat of HCV transmission through blood transfusions [70].

Together, these diagnostic and preventive strategies serve as the foundation for global HCV control efforts. Continued refinement and equitable expansion of these programs remain central to achieving long-term elimination goals.

## 9. Economic and Health Outcomes of Hepatitis C Screening and Treatment Programs

The elimination of HCV as a public health threat by 2030, a target set by the WHO, requires not only epidemiologically validated strategies but also economically sustainable interventions. Health economics and outcomes research (HEOR) approach is essential for assessing the cost-effectiveness, budget impact, and long-term social value of screening, diagnostic, and treatment strategies for HCV. As more countries expand access to testing and DAA therapy, integrating economic analyses will become crucial for informing evidence-based public health policy decisions. Multiple studies have demonstrated that HCV elimination is not only clinically feasible but also cost-effective in diverse settings. A global modeling study indicates that expanding prevention and treatment interventions could prevent 15.1 million new infections and 1.5 million deaths by 2030, resulting in substantial healthcare savings [71].

Recent national-level evaluations further support these findings. In South Korea, Choi and his colleagues demonstrated that universal screening of adults aged 40–65 years, followed by DAA treatment, is highly cost-effective, well below the national willingness-to-pay threshold [68]. In Europe, a multicenter analysis conducted in Spain showed that large-scale treatment is cost-effective even in countries with low prevalence, particularly when combined with decentralized screening models and simplified care pathways [67].

From a healthcare-system perspective, integrating HCV testing into emergency departments, prisons, and primary care represents a strategic opportunity for early case identification by leveraging existing infrastructure. An evaluation conducted in the UK demonstrated that “opt-out” BBV testing in emergency services not only increased diagnostic rates but also proved to be cost-saving in the medium term due to early linkage to treatment [72].

Moreover, economic evaluations also highlight the benefits regarding health equity. “Test-and-treat” strategies targeting disadvantaged populations not only improve clinical outcomes but also reduce health disparities, supporting the inclusion of social justice principles in cost-effectiveness analyses [73]. These conclusions are perfectly aligned with WHO priorities on universal health coverage and equitable access to care [74].

## 10. Discussion

For many years, Egypt has been considered one of the countries most heavily affected by HCV infection, historically reporting the highest global prevalence rates. The widespread introduction of DAA therapies has significantly transformed the national response to this public health crisis. These effective treatment regimens have enabled notable progress in Egypt’s HCV control strategy, substantially reducing the disease burden. This success underscores how sustained political support, strategic planning, and broad treatment access can drive national-level epidemic control [75].

In line with the WHO targets for HCV elimination—diagnosing at least 80% of infected individuals and treating 70% of those diagnosed—Egypt has surpassed these benchmarks. The national screening campaign identified approximately 87% of those with HCV, with 93% receiving curative antiviral therapy. Consequently, more than four million individuals with chronic hepatitis C have been successfully treated, making Egypt the first country to receive WHO validation for being on the path toward HCV elimination. This achievement underscores the strength of Egypt’s healthcare system and provides a model for other nations aiming to integrate screening, treatment, and policy interventions [75].

Across Europe, various elimination strategies have emerged at both local and national levels. The French Ministry of Health piloted a test-and-treat program in Perpignan which subsequently guided nationwide adoption. Similarly, the Hep Care project, led by University College Dublin with European Union (EU) funding, showed that targeted micro-elimination programs can reduce HCV prevalence among high-risk groups within multiple European contexts. These initiatives underscore the importance of adapting local approaches within comprehensive elimination frameworks [76].

Spain has also made substantial progress toward WHO’s HCV elimination targets, which include an annual incidence of five or fewer cases per 100,000 people, two or fewer per 100 among PWIDs, and HCV-related mortality rate limited to a maximum of two deaths per 100,000 individuals. Achieving these goals depends on consistent implementation of prevention, high diagnostic coverage, and widespread treatment. Spain’s coordinated efforts align with global targets and offer valuable insights for other countries [77,78].

Correctional facilities are a focal point in WHO’s HCV elimination plan, as people in prison are at significantly higher risk—often tenfold—compared to the general population. This increased vulnerability is primarily linked to behaviors such as IDU. HCV continues to pose a major health challenge in institutional settings, with recent figures indicating a prevalence of 14.8% in facilities under the Ministry of the Interior and nearly 12% in prisons across Catalonia. In recent years, these prevalence rates have declined, coinciding with reduced HIV incidence and lower levels of substance use among this population, likely influenced by prevention programs and expanded antiviral-treatment access [79].

The high turnover and movement of detained individuals between prisons and communities contribute to the risk of HCV spread. Effective prison-based screening and treatment represent essential components of comprehensive public health strategies. Treating this population reduces overall transmission and supports national and global elimination efforts [77,80].

Following the introduction of DAA therapy in Catalonian prisons starting in 2015, more than 1000 incarcerated people have successfully reached SVR. However, reinfection remains a challenge among individuals who continue engaging in high-risk behaviors, especially IDU, as viral clearance does not confer immunity. Ongoing risk reduction strategies and reinfection monitoring are thus vital for maintaining treatment success [77].

A study by Cambianica et al. investigated the hepatitis C care pathway within two penitentiary institutions located in Brescia, Northern Italy. The findings confirmed higher HCV seroprevalence among people in detention than in the general population and revealed poor screening adherence. The authors recommended enhanced education and test-and-treat strategies to improve engagement and outcomes [69].

Another study, led by Saludes and colleagues, conducted across eight Catalan prisons, showed low reinfection rates following DAA treatment. Nevertheless, reinfection risk persists, particularly among newly incarcerated individuals or those from vulnerable groups, including people living with HIV, homeless individuals, and PWIDs. Prisons offer a valuable opportunity to deliver care to populations often excluded from mainstream services, thus also contributing to community-level transmission reduction [77].

In the Catalan study, over 80% of participants reported having injected drugs at some point, and 28% of viremic individuals had done so during prior incarcerations. Among those monitored for reinfection, one-third continued intravenous drug use during treatment. These findings reaffirm that IDU remains the main driver of ongoing HCV transmission in correctional settings globally. Integration of harm-reduction services with medical treatment is critical in mitigating these risks [77].

Global differences in prison reinfection rates stem not only from local HCV prevalence but also from the public health models used. Effective harm-reduction strategies play a critical role in this context. Needle exchange programs and opioid substitution therapy within prisons are recognized as key measures for reducing reinfection and supporting sustained treatment success [77,81].

In its national strategy for eliminating HCV, Canada has designated individuals with a history of incarceration as a priority group for intervention. They account for roughly 10% of the national HCV burden. According to the recent data from the Public Health Agency of Canada, an estimated 10% of incarcerated individuals—equivalent to approximately 38,000 people—have had prior exposure to HCV. This highlights the urgent need to improve access to testing and medical care within correctional institutions. The frequent movement between prison and community settings further highlights the importance of comprehensive prison-based care in achieving national elimination goals [51,82,83,84,85,86,87].

An estimated 55,354 individuals in San Diego County, California, have been infected with HCV, underscoring the region’s significant epidemiological burden. In 2018, the Owen Clinic at the University of California San Diego launched a targeted micro-elimination program focused on managing HIV-HCV co-infection among vulnerable groups. This initiative later developed into the “Eliminate Hepatitis C Initiative”, a collaborative effort between public institutions and the American Liver Foundation. Formalized in 2021, the plan aligns with WHO goals by promoting awareness, screening, linkage to care, and treatment throughout the care continuum [88,89].

To achieve an 80% reduction in HCV incidence, modeling suggests that without expanded harm-reduction measures, treatment coverage must reach 60% annually among HCV-positive individuals without HIV. These findings stress the need for combining scaled-up therapy with interventions that reduce transmission risk [88].

Research led by Wedemeyer et al. found that treating 10% of infected individuals annually could lead to a 90% reduction in HCV infections by 2030. Large-scale implementation of high-SVR antiviral regimens holds great potential to reduce deaths and disease burden. Even modest improvements in diagnosis and treatment can significantly reduce the disease burden [90].

In Mexico, a study by Jose Abrego et al. reported an HCV prevalence rate of 36.4% among individuals diagnosed with HIV. The primary transmission route was IDU, linked to intense drug trafficking and high incarceration rates in the western region [91].

Taiwan introduced an HCV screening and treatment initiative targeting the incarcerated population at Yulin Prison. Individuals testing positive for antibodies were promptly treated with DAAs, achieving nearly 100% SVR. The program’s success underscores the importance of collaboration between correctional and medical staff [92]. For individuals with a history of IDU, OAT remains a cornerstone of care. An on-site “test-and-treat” approach was developed, integrating rapid HCV/HIV screening with prompt initiation of DAA therapy. Over three years, the program reduced HCV prevalence from 38% to 7%, highlighting the impact of targeted prison-based interventions [5,93].

Recent findings from both qualitative and interventional research highlight key challenges and opportunities in addressing hepatitis C among PWIDs. In rural areas of Northern New England, limited access to sterile syringes and significant gaps in the understanding of HCV transmission, diagnosis, and treatment have been linked to high-risk behaviors such as syringe sharing, underscoring the need for expanded education and harm-reduction services [94]. Meanwhile, a hospital-based trial in Oslo, Norway, demonstrated that initiating HCV treatment as part of inpatient care can reduce time to viral clearance, offering a promising strategy for engaging marginalized populations who may otherwise drop out of care [95]. However, persistent reinfection—particularly among PWIDs—reinforces the importance of integrating treatment with robust, sustained prevention efforts [94,95].

Integrating clinical interventions with an understanding of community-level risk factors is essential. Recent studies emphasize this complementary: one demonstrates the success of inpatient treatment strategies [94], while the other reveals vulnerabilities on the ground, particularly in rural areas.

In Australia, a study by Scott et al. highlighted the need to significantly increase HCV testing to meet WHO elimination goals. The study estimated that testing coverage must grow by at least 50% to capture undiagnosed cases and halt transmission [96].

A similar study by Gountas and colleagues in Greece concluded that large-scale screening programs are essential for elimination. Passive detection and low awareness lead to undiagnosed cases and advanced disease, making them economically inefficient. Comprehensive elimination strategies are more cost-effective by preventing long-term outcomes [97].

Several studies from various global regions, including correctional facilities, have evaluated progress toward HCV elimination. While some countries are on track to meet WHO benchmarks, others face structural or epidemiological challenges.

Table 3A,B summarize studies evaluating HCV elimination efforts at national and local levels. For clarity, studies were organized according to the level of intervention: macro-level national strategies (Table 3A) and targeted micro-elimination initiatives in high-risk populations (Table 3B). This structure underscores the heterogeneity of outcomes influenced by policy frameworks, healthcare infrastructure, and population risk profiles. Such comparative evidence is relevant for guiding future implementation strategies adapted to different healthcare settings.

While Table 3A focuses on national-level strategies and policy-oriented interventions, Table 3B highlights micro-elimination approaches implemented in high-risk or underserved populations. These targeted strategies are essential complements to national programs, as they address persistent transmission pockets and improve equity in access to HCV diagnosis and treatment.

The exclusive focus of current strategies on vulnerable populations should be complemented by screening measures targeting the general population, as this is crucial for a comprehensive and sustainable approach to HCV elimination. Several countries have already adopted such integrated models. According to the Centers for Disease Control and Prevention (CDC) guidelines in the US, all adults should receive a one-time HCV test, and people born between 1945 and 1965 should be screened more regularly [108]. Evidence consistently demonstrates that these approaches significantly increase diagnostic and treatment initiation rates while remaining cost effective in the era of highly effective direct-acting antivirals [71]. Notably, cohort-based testing has been shown to perform comparably to risk-based testing in identifying infected individuals, while offering greater feasibility for large-scale, system-wide implementation [109]. These findings emphasize the practical feasibility and public health value of integrating population-level screening into routine healthcare services.

To eliminate hepatitis C at national and global levels, strategic frameworks must prioritize universal access to DAAs and the integration of robust prevention efforts. These include systematic screening, safe medical practices, harm-reduction services, and public health education to combat stigma. A coordinated approach combining early diagnosis, immediate treatment, and prevention is the most effective path to ending HCV as a public health threat [110].

## 11. Remaining Barriers and Knowledge Gaps in HCV Elimination

Despite important progress in screening, diagnosis, and treatment, several knowledge gaps continue to limit the optimization of HCV elimination strategies. Data on hard-to-reach groups, such as PWIDs, prisoners, and migrants, are often incomplete or inconsistent, making it difficult to design effective programs. Long-term studies on reinfection rates, treatment adherence, and the population-level impact of DAA therapy are still limited. Research on cost-effectiveness is also limited in LMICs, where decisions on how to use resources are crucial. The integration of HCV screening and treatment into primary healthcare is poorly documented, and digital tools for patient follow-up and linkage to care are rarely used. Closing these gaps will require coordinated international studies, improved patient registries, and outreach strategies adapted to local epidemiological and socioeconomic conditions [46,111,112]. The analysis of national strategies illustrates how these gaps translate into context-specific barriers (Table 4). In Egypt, although mass testing campaigns achieved very wide coverage, important challenges were reported, including the initial stigmatization of the population and the high logistical costs required for large-scale implementation [113]. In Spain, the inclusion of screening within primary healthcare services was limited by a lack of human resources and marked regional differences, which affected the consistency and effectiveness of the program [98,114]. In Australia, while the universal treatment program was an important step in improving access to care, it continued to struggle to reach marginalized groups, particularly PWIDs [115]. In all three settings, financial sustainability and the continued implementation of prevention campaigns remain shared and significant challenges, highlighting the need for coordinated and durable public health strategies.

## 12. Conclusions

The elimination of HCV infection remains an important global public health goal, supported by major progress in diagnosis, treatment, and prevention. The availability of DAA therapies—effective and well-tolerated—has changed how the disease is managed, turning hepatitis C into a curable and potentially eliminable infection.

A key part of this effort is the wide use of screening programs that help detect infections early, especially in high-risk groups. These include PWIDs, those who had blood transfusions before routine screening was introduced, people in prison, and underserved communities with limited access to healthcare. Finding and treating cases in these groups is essential to reducing the spread of the virus and to increasing access to therapy.

To reach elimination targets, screening must be matched by equitable and sustained access to treatment. Everyone affected—regardless of income, stage of illness, or personal risk factors—should be able to receive antiviral therapy. This helps reduce health inequalities and supports progress at both national and global levels.

It is also important to keep monitoring risk factors and to develop long-term, practical public health strategies. Utilizing digital tools such as patient tracking systems to improve follow-up and program planning can help increase the effectiveness of these efforts, and significant barriers and knowledge gaps still limit progress.

Despite these advances, HCV elimination is still limited by significant barriers and knowledge gaps. Disparities in healthcare infrastructure, funding sustainability, and social acceptance continue to restrict program expansion, while hard-to-reach populations remain insufficiently engaged. Robust data on reinfection rates, long-term adherence to antiviral therapy, and cost-effectiveness in LMICs are still lacking. Successful national programs in Egypt, Spain, and Australia show that political will and financial investment alone are not enough. Tailored interventions adapted to local epidemiological and socioeconomic contexts, targeted research, and stronger global cooperation are essential to overcoming these barriers and improving program effectiveness.

This review aims to support the foundation of prevention and control initiatives by offering a comprehensive analysis of the most relevant aspects related to the epidemiology, diagnosis, treatment, and prevention of HCV infection. By synthesizing current evidence and outlining the necessary strategic directions, this work contributes to the global coordination efforts required to transform the elimination of hepatitis C from an aspirational goal into a tangible achievement with meaningful impact on worldwide public health. Sustained global commitment and coordinated implementation of these strategies are essential to achieving this public health milestone.

## 13. Future Directions

### 13.1. Telemedicine—Expanding Access to Diagnosis and Treatment

Limited access to specialized healthcare services remains a major obstacle to the elimination of HCV, particularly for individuals living in remote areas or belonging to vulnerable groups. In this context, telemedicine represents a promising strategy to facilitate access to care and ensure continuity throughout the diagnostic and treatment process.

Remote consultations can support the initial evaluation of individuals at risk for HCV infection by enabling the interpretation of laboratory results and directing further investigations. Moreover, they allow real-time monitoring during DAA therapy, enabling timely treatment adjustments in response to therapeutic efficacy and adverse effects.

Beyond clinical management, digital platforms can provide psychological support and educational content through online sessions that improve adherence and support successful treatment outcomes. A practical example is the implementation of structured hepatology teleconsultation programs, in collaboration with general practitioners, which may shorten the time between diagnosis and treatment initiation by removing geographic and logistical barriers. This approach enhances interdisciplinary communication, supports shared decision-making, and ensures secure transfer of medical data throughout the care pathway [116].

### 13.2. Medical Informatics—Optimizing Data Management and Patient Flow

The digitalization of healthcare systems offers a strong foundation for streamlining clinical workflows and enabling long-term follow-up of patients with HCV infection. Electronic registries of diagnosed cases, accessible to primary care providers, specialists, and public health authorities, can support coordinated monitoring and allow for timely interventions in cases of treatment discontinuation or non-adherence [117].

Automated alert systems embedded in electronic health records (EHRs) can flag abnormal laboratory values indicative of possible HCV infection, prompting confirmatory testing. These tools help reduce delays in diagnosis and prevent loss to follow-up [118].

Furthermore, artificial intelligence algorithms applied to clinical, laboratory, and demographic data can identify individuals at higher risk of infection. This predictive approach may enhance the targeting of screening interventions and promote more efficient use of healthcare resources [119].

A concrete example is the integration of alert modules into EHR systems that identify patients with elevated liver enzymes, a history of transfusions, or other relevant risk factors. These tools have proven effective in increasing testing uptake and accelerating access to antiviral therapy [120].

### 13.3. Expanding Screening Programs for High-Risk Populations

One of the central strategic directions in the elimination of hepatitis C is the expansion of screening programs, particularly targeting high-risk populations and individuals with limited access to healthcare services. Future interventions should include universal screening in high-prevalence settings, such as hospitals, emergency departments, mental health institutions, and correctional facilities [74,121]. The deployment of mobile testing units and the implementation of community-based screening initiatives can significantly improve coverage in underserved or geographically isolated areas. Furthermore, the integration of digital algorithms and EHRs can enhance logistical efficiency and facilitate the early identification of high-risk individuals.

For such interventions to be effective and fair over the long term, they must align with the social and cultural context of the target communities. They should also uphold data confidentiality and promote stigma-free environments. Overcoming barriers like inadequate healthcare infrastructure, low health literacy, and interruptions in care demands an integrated response that brings together multiple sectors and engages local partners. Beyond the clinical benefits, early detection and timely treatment can generate substantial long-term cost savings by reducing the burden associated with advanced liver disease.

### 13.4. Public Awareness and Health Education Campaigns

Educational and awareness initiatives targeting the general public play an essential supporting role in efforts to eliminate hepatitis C. These campaigns contribute to increased awareness of transmission risks, the importance of early testing, and the availability of effective antiviral therapies. Communication strategies must be clear, accessible, and culturally sensitive, targeting both the general population and vulnerable high-risk groups.

Reducing hepatitis C-related stigma is a key objective. This can be achieved through the ongoing training of healthcare professionals, fostering empathetic and non-discriminatory behavior within healthcare settings, and incorporating hepatitis C-related content into medical education curricula. Targeted outreach campaigns, developed in partnership with community leaders and NGOs, can promote engagement and support the integration of affected individuals into healthcare systems.

### 13.5. Alignment with WHO Strategies and Recommendations

To ensure the effectiveness and sustainability of hepatitis C elimination efforts, national public health policies must be aligned with the strategic guidelines issued by the WHO. This framework involves ensuring universal access to testing and treatment, establishing strong harm reduction programs, especially for PWIDs, and enhancing surveillance systems to effectively track progress toward elimination goals.

An essential component of this alignment is the continuous training of healthcare professionals to provide non-discriminatory, patient-centered care. Public awareness and education campaigns must also be sustained, using digital platforms and social media to disseminate culturally appropriate messages. The success of these efforts depends on multisectoral collaboration involving public health authorities, NGOs, the private sector, and affected communities.

Such an integrated and coordinated approach represents a critical pillar in achieving global hepatitis C elimination goals.

### 13.6. Ensuring Universal Access to Antiviral Treatment

Building on this strategic framework, ensuring equitable and universal access to DAA therapies remains a central operational priority. Removing financial and administrative barriers is essential to increasing treatment uptake, particularly among vulnerable and hard-to-reach populations. This includes engaging in negotiations at the national or regional level with pharmaceutical manufacturers and supporting the production and distribution of generic medicines in LMICs [122,123].

The simplification of how treatment is initiated represents an equally critical aspect. Standardized treatment protocols applicable in both primary care and community settings can facilitate rapid diagnosis and early identification of patients eligible for therapy [71]. Coverage for essential diagnostic procedures—such as HCV RNA testing and viral genotyping—through reimbursement mechanisms further supports treatment adherence and patient engagement.

The elimination of unjustified administrative restrictions, such as treatment eligibility criteria based on liver disease stage or past substance use, is critical for promoting equitable access to care [124,125]. In parallel, scaling up early testing efforts among high-risk populations can accelerate case detection and reduce community-level transmission. Providing treatment in a supportive, respectful setting helps reduce stigma around HCV and reinforces the message that it is a curable disease and that affected individuals deserve care, dignity, and inclusion. Embedding these principles into national public health strategies is essential to achieving global hepatitis C elimination targets.

### 13.7. Supporting Collaborative Efforts Across Countries and the Adoption of Evidence-Based Practices

Enhancing international partnerships and facilitating the dissemination of effective intervention models are essential elements of the global strategy for eliminating HCV. Given the significant disparities in healthcare infrastructure across regions, the practical experience accumulated by countries such as Egypt and Australia—both of which have made notable progress in this area—can guide the implementation of adapted solutions in resource-limited settings [96,113].

Broad access to validated therapeutic protocols, clinical guidelines, educational materials, and modern technologies depends on close cooperation with leading international organizations, such as the WHO, the CDC, and the ECDC [19,126,127]. Such collaborations support standardized care while enabling adaptation to each country’s unique epidemiological and social context.

Furthermore, creating a unified international structure, possibly guided by WHO, to track progress in HCV elimination could allow for the systematic collection and comparison of data related to national plans and their practical application. Such a mechanism would support continuous evaluation of intervention effectiveness and encourage the use of tested models, promoting evidence-based approaches. Constant communication between countries, supported through such a structure, could accelerate global efforts and help reduce inequalities in regard to access to diagnosis and treatment.

### 13.8. Advancing Vaccine Development for Hepatitis C

Advancing vaccine development for HCV continues to represent a critical objective in ongoing biomedical research efforts. Although antiviral therapies have achieved remarkable progress, there is still a need for a preventive solution that reduces the risk of transmission and supports eradication efforts. The main challenges in this area arise from the virus’s high genetic diversity and its ability to evade the host immune response.

Future research is focused on identifying viral components with high immunogenic potential, capable of eliciting a durable and effective immune response. In parallel, innovative vaccination technologies—such as messenger RNA (mRNA) platforms and viral vectors—are being explored, offering the possibility to tailor immune responses and enhance protection.

A deeper understanding of how HCV interacts with the immune system is essential for the development of viable prophylactic strategies. Studies on viral persistence mechanisms and immunological correlates of protection are expected to contribute to the optimization of vaccine candidate design.

Integrating these research directions outlines the possibility of developing a safe, effective, and broadly applicable vaccine. Such an innovation would complement existing therapeutic interventions and represent a valuable tool in the long-term control of HCV infection.

## Figures and Tables

**Table 1:** Identified risk factors for HCV infection.

No.	Author(s) and Year	Study Type	Identified Risk Factors	Studied Population	Quality of the Study	Key Conclusions
1.	Meteliuk et al., 2024 [32]	Cohort (retrospective observational)	Reduced access to opioid substitution therapies; increased high-risk behavior in conflict zones	Patients receiving opioid substitution therapy in Eastern Ukraine, and Crimea	Moderate quality: limited generalizability beyond conflict settings; potential selection bias	The 2014 military conflict reduced enrollment and retention in opioid agonist therapy (OAT), increasing HCV transmission risk among PWIDs
2.	Bashir et al., 2022 [33]	Cross-sectional (descriptive)	Lack of proper sterilization practices beauty salons	Beauty salon worker in Karachi, Pakistan	Moderate quality: small, localized sample; self-reported practices may introduce reporting bias; cannot interfere causality	Lower awareness and unsafe practices regarding HBV and HCV prevention were identified, highlighting the need for education and training programs to reduce transmission risks in these settings
3.	Caminada et al., 2023 [34]	Case–control (observational)	Exposure to various invasive procedures and occupational risks associated with increased HBV and HCV infection	8176 cases of acute hepatitis B; 2179 cases of acute hepatitis C, compared with hepatitis A controls	Moderate quality: potential recall bias; possible confounding due to unmeasured variables	The study highlighted an increased risk of viral hepatitis infections linked to invasive procedures, underscoring the need for enhanced infection prevention protocols in healthcare settings
4.	Epstein et al., 2018 [35]	Cohort (observational)	Opioid use during pregnancy; maternal HIV co-infection	Pregnant women with HCV and their infants	Moderate quality: incomplete infant follow-up; underestimation of true perinatal transmission; reporting and selection biases	The perinatal HCV transmission rate was 2.8%; only 45% of exposed infants completed recommended screening, and just 41% of viremic mothers were linked to postnatal HCV care, revealing critical gaps in the care continuum
5.	Newsum et al., 2021 [36]	Cohort (Prospective longitudinal)	High-risk sexual behaviors and HIV co-infection	HIV-positive MSMs	Moderate quality: risk behaviors self-reported; limited to HIV-positive MSMs, reducing external validity	The HCV reinfection rate was 11.5 per 100 person-years and was strongly associated with high-risk sexual behaviors
6.	Monin et al., 2023 [37]	Cohort	HIV co-infection	HIV-positive MSMs with recent HCV infection	Moderate quality: small sample size; short follow-up duration	The low rate of spontaneous HCV clearance highlights the importance of early antiviral treatment to prevent disease progression and reduce transmission risk
7.	Terrault et al., 2013 [38]	Cohort	HCV-positive partner in monogamous heterosexual relationships	Monogamous heterosexual couples with one HCV-positive partner	Moderate quality: low event rate limits statistical power; older data may not reflect current risk behaviors; potential recall and selection biases	HCV sexual transmission was very low (~0.07% per year), indicating negligible risk

**Table 2:** HCV screening approaches and challenges in various populations.

No.	Author(s) and Year	Study Type	Country/Region	Screening Method	Studied Population	Key Findings	Identified Challenges
1.	Cuomo et al., 2019 [56]	Retrospective	Italy (Moderna)	Serological testing for HBV, HCV, HIV, syphilis; Mantoux test and chest X-ray for tuberculosis (TB)	304 recent migrants	Detected high infection rates: HBV (12.2%), HCV (3.3%), HIV (1.6%), syphilis (0.7%), and latent TB (10.2% positive Mantoux; 6 active TB cases confirmed); findings support the need for universal infections disease screening and early intervention strategies in migrant populations	High infections disease burden, delayed healthcare access, and logistical challenges in follow-up and treatment
2.	Evans et al., 2018 [57]	Prospective observational	UK (London)	Opt-out serological testing for HBsAg and HCV antibodies, with confirmatory HCV antigen testing	3290 adult patients attending an emergency department	HBsAg prevalence of 0.5%, HCV antibody prevalence of 2.0%, HCV antigen prevalence of 1.2%. Risk factors: male gender, non-White British ethnicity, homelessness. Linkage to care: Achieved in 93% of HBV-positive, and 78% for HCV-positive patients	Limited access to healthcare, lack of awareness, unstable living conditions
3.	Monti et al., 2025 [58]	Prospective	Italy (Tuscany)	On-site rapid finger-prick testing for HBsAg and anti-HCV	1812 individuals from marginalized communities, recruited via Non-Governmental Organizations (NGOs) outreach	HCV antibody positivity was 2.9%; linkage to care was achieved in 37.8% of those testing positive; all patients with confirmed viremia received and completed successful treatment	Highlights the need for tailored outreach strategies to improve screening uptake and linkage to care among underserved populations
4.	Pinazo-Bandera et al., 2024 [59]	Prospective	Spain (Malaga)	Dry drop test (DDT) for HCV antibodies, followed by confirmatory diagnostics and treatment in a single hospital visit	417 individuals from vulnerable populations (PWIDs and homeless individuals) invited; 271 (65%) participated	HCV antibody positivity was 10%; 23 patients-initiated treatment with DAAs; SVR was achieved in 96%	Limited healthcare access, lack of awareness, and unstable living conditions
5.	Barror et al., 2019 [60]	Prospective multisite feasibility	Ireland, UK, Romania, Spain	Community-based intensive screening including HCV antibody and RNA testing, liver fibrosis assessment, and referral to care	2822 individuals recruited from drug treatment centers, homeless shelters, and prisons	19% had active HCV infection; 80% of those diagnosed were successfully linked to specialized care	Difficulty in engaging vulnerable populations; barriers in ensuring consistent and effective linkage to care
6.	Strehlow et al., 2012 [61]	Cross-sectional (observational)	USA	Serological testing for anti-HCV	387 homeless adults attending Health Care for the Homeless clinics	HCV prevalence was high (31%, with a markedly higher rate among PWIDs (70%); over half (53.3%) were unaware of their infection status	Limited healthcare access, and stigma as key barriers to HCV diagnosis and care
7.	Gelberg et al., 2012 [62]	Cross-sectional	USA (Los Angeles) California	Serological testing for anti-HCV	534 homeless adults recruited from shelters and food programs	HCV prevalence was 26.7%, primarily associated with IDU; 46.1% of infected individuals were unaware of their status	Limited healthcare access, low education, incarceration history, and socioeconomic vulnerability
8.	Khalili et al., 2022 [63]	Prospective	USA (San Francisco, Minneapolis)	Rapid anti-HCV and HCV RNA testing	766 homeless adults from four urban shelters	HCV prevalence was 21.1%, with 66% having active infections; nearly half were unaware of their status. An integrated, shelter-based care model enabled treatment initiation in 61.7% of identified cases	Limited healthcare access, low awareness, substance use, and psychiatric comorbidities
9.	Pereira et al., 2013 [64]	Cross-sectional	Brazil	Serological testing for anti-HCV	19,503 individuals aged 10–69 years	National HCV prevalence was 1.38%, and it was higher among older adults; key risk factors included IDU, blood transfusions, hospitalizations, and tattoos	Low awareness, limited healthcare access, and inadequate sanitation infrastructure
10.	Coppola et al., 2020 [65]	Prospective	Italy (Southern Italy)	Serological testing for HBsAg, anti-HCV, and anti-HIV	3839 adult immigrants from seven clinical centers	Prevalence of HBV (9.9%), HCV (3.5%), and HIV (1.6%); prevalence differed by gender, age, and region of origin	Disparities by socio-demographic and regional factors, with limited availability of healthcare
11.	Segala et al., 2024 [66]	Cross-sectional	Italy (Apulia)	Serological testing for HIV, HBV, HCV, and syphilis	149 individuals, including 64 migrant agricultural workers, and 85 homeless persons)	9.4% tested positive for HCV; only 50.3% of the screened individuals collected their tests results	Very limited access to healthcare services (only 14.1% of migrants had access to primary care); low awareness of sexually transmitted infections (STIs) and engagement in high-risk sexual behaviors

**Table 3:** (**A**) Macro-level national strategies for hepatitis C elimination. (**B**) Micro-elimination initiatives targeting high-risk populations.

(**A**)					
**No.**	**Author(s) and Year**	**Study Design**	**Country/Region**	**Objectives**	**Key Findings**
1.	Calleja et al., 2023 [98]	Theoretical Modeling (dynamic transmission + cost-effectiveness)	Spain	Assessment of prevalence, incidence, and cost-effectiveness of expanded testing and treatment strategies for hepatitis C	Using a disease transmission model and cost analysis, results show that scaling up these measures can significantly reduce HCV prevalence and incidence, while being economically efficient in achieving elimination goals
2.	Feld et al., 2022 [99]	Theoretical Modeling (HCV elimination scenarios)	Canada	To assess when Canadian provinces are expected to eliminate HCV and to identify the strategies needed to achieve this goal	Without increasing efforts, Manitoba, Ontario, and Quebec projected to miss HCV elimination target by 2030. Timely elimination in these provinces could prevent 170 deaths and save about 122.6 million Canadian dollars in direct medical costs. The study estimated specific annual treatment targets required to meet elimination goals
3.	Snell et al., 2023 [100]	Descriptive	Canada	Analysis of public reimbursement policies for DAAs for HCV in Canada and their impact on treatment access	Significant variations in eligibility criteria and restrictions across provinces and territories were identified, potentially leading to inequities in access to treatment and undermining efforts toward national HCV elimination
4.	Brouard et al., 2019 [101]	Cross-sectional	France	Assessment of HCV and HBV prevalence in the general population in France using self-collected blood samples and screening history	In 2016, the prevalence of chronic HCV infection was 0.30%, with 80.6% of infected individuals already aware of their status. These findings contributed to shaping the foundation of France’s new national screening strategy
5.	Brouard et al., 2020 [102]	Cross-sectional	France	To assess the HCV care cascade in France before and after the introduction of DAAs, evaluating their impact on diagnosis, care, and treatment rates	Between 2011 and 2016, the number of individuals with chronic HCV infection in France decreased by 31%, from approximately 192,700 to 133,500. Awareness of infection among those affected increased from 57.7% to 80.6%. However, by 2016, only 25.7% of infected individuals were receiving care, and 12.1% were undergoing treatment, indicating that despite the positive impact of DAAs, significant gaps remained in accessing care and treatment
6.	Safreed-Harmon et al., 2018 [103]	Cross-sectional	Nordic countries (Denmark, Finland, Iceland, Norway, Sweden)	Evaluation of policy responses to HCV in the Nordic region and identification of reporting gaps and discrepancies	Iceland was the only country with a national HCV elimination strategy. Availability of harm reduction services varied across countries, and notable discrepancies were found in reports—particularly from government institutions and NGOs. The study highlights the need for better coordination and clearly defined national strategies to support HCV elimination efforts across the region
7.	Drose et al., 2022 [104]	Implementation (observational)	Denmark	Multi-level HCV elimination plan, Southern Denmark, by 2025	Target: 90% HCV diagnosis and 80% treatment coverage by 2025, five years ahead of the WHO target. An estimated 3028 individuals in the region were HCV-RNA positive, with 33% in care, 43% diagnosed but not in care, and 24% undiagnosed. Five interventions: expanded testing and treatment in addiction care and correctional settings, recontacting patients lost to follow-up, and improved surveillance systems
8.	Whittaker et al., 2024 [105]	Theoretical Modeling	Norway	To assess Norway’s progress toward eliminating HCV, with a focus on PWIDs and immigrants	The model estimated that in 2022, there were 30 new HCV infections among active PWIDs—a sharp decline from a peak of 726 cases in 2000. An estimated 3202 individuals were living with chronic HCV in 2022. These findings highlight the effectiveness of Norway’s harm reduction services and unrestricted treatment policies in driving progress toward elimination
(**B**)					
**No.**	**Author(s) and Year**	**Study Type**	**Country/Region**	**Objectives**	**Key Findings**
9.	Cambianica et al., 2024 [69]	Retrospective (observational)	Italy (Brescia)	To evaluate HCV screening, diagnosis, and treatment among prisoners in two large penitentiaries	Only 54.5% were screened; 9.2% were HCV antibody positive. Of 169 RNA-positive, 77 were treated, with high cure rates. Post-release care continuity was poor
10.	Saludes et al., 2023 [77]	Prospective	Spain (Catalonia)	To determine incidence and molecular epidemiology of HCV reinfection among incarcerated individuals in Catalonia, focusing on those previously treated and those entering prison	Among newly incarcerated individuals, 2% were viremic, with 13.5% representing reinfections, mostly among PWIDs. Phylogenetic analyses showed that viral strains in prisons closely resembled those in the general population, suggesting interconnected transmission pathways
11.	Kronfly et al., 2021 [106]	Cross-sectional	Canada	To assess how HCV testing, therapy, and harm reduction measures are implemented in adult provincial correctional facilities in Canada	HCV care varied widely across Canadian provincial prisons. Only 54% had ever initiated treatment, and screening was inconsistent, with some offering no screening at all. Limited access criteria; no structured care linkage in place. Prisons under health ministry oversight offered better services. The study highlights the need for standardized opt-out screening and broader treatment access
12.	Mambro et al., 2024 [82]	Qualitative	Canada (Quebec)	To explore how individuals with incarceration experience perceive HCV and its management, aiming to understand factors influencing engagement in care	Among 19 participants with a history of HCV infection and incarceration, perceptions were influenced by fears of transmission, death, family impact, and stigma (HCV, IDU, and incarceration). Coping strategies varied: some participants turned to education and support networks, while others engaged in self-isolation or high-risk behaviors. Despite advancements in HCV treatment, stigma and fear continue to limit timely care engagement among this population
13.	Yang et al., 2020 [107]	Prospective cohort	Taiwan	The study aimed to assess the feasibility and impact of a micro-elimination strategy for chronic HCV infection among incarcerated individuals in Taiwan. This strategy involved universal HCV screening followed by treatment with DAAs	The implementation of universal HCV screening and subsequent DAA strategy among the incarcerated population in Yunlin Prison led to a significant reduction in HCV prevalence. The study demonstrated that such a micro-elimination approach is both feasible and effective in a correctional setting, contributing to the broader goal of HCV elimination
14.	Bregenzer et al., 2022 [93]	Cohort	Switzerland	The study aimed to evaluate the feasibility and outcomes of an HCV elimination strategy within an OAT program. This involved systematic HCV screening and treatment with DAAs	The introduction of systematic HCV screening and DAA therapy in the OAT setting significantly reduced HCV prevalence. Micro-elimination was practical and successful in routine care, supporting HCV elimination efforts
15.	Romo et al., 2024 [94]	Qualitative	USA (rural Northern New England)	To explore HCV risk factors among PWIDs, focusing on syringe access, sharing behaviors, and perceptions of HCV	Limited syringe access led to sharing, decisions where shaped by perceived HCV risk, personal trust, and misinformation. There was widespread confusion about HCV transmission, testing, and treatment
16.	Midgard et al., 2024 [95]	Cluster randomized trial	Norway	To evaluate the effectiveness of an opportunistic test-and-treat approach for HCV infection among hospitalized PWIDs, comparing immediate treatment initiation during hospitalization to standard outpatient referral	Treatment completion was higher with in-hospital initiation. Faster treatment initiation; cure rates unchanged. The opportunistic approach outperformed standard care
17.	Abrego et al., 2024 [91]	Cross-sectional	Mexico (western region)	Assessed HCV prevalence, risk factors, genotypes, and fibrosis in HIV-infected patients	HCV co-infection was present in 36.4% of HIV patients, IDU, history of incarceration, early sexual activity, blood transfusions, tattooing, sex work, and surgery were identified as significant risk factors. Most common: genotype 1a (68.2%). Advanced liver fibrosis in 47.7% of co-infected; low CD4, low albumin, and high bilirubin

**Table 4:** Comparative analysis of the barriers and limitations of national strategies.

Country	Main Type of Intervention	Key Barriers/Limitations	Lessons Learned
Egypt [113]	Nationwide mass screening and free DAA treatment	High logistical and financial costs Initial stigma	Community engagement and education campaigns are essential to overcome stigma Strong political commitment and stable funding enable large-scale programs
Spain [98,114]	HCV screening integrated into primary healthcare and micro-elimination programs	Limited human resources in primary care Regional variability in implementation	Standardized national guidelines improve consistency Training primary care staff improves case finding and care linkage
Australia [115]	Universal access to DAA therapy through national reimbursement programs	Difficulty reaching marginalized populations (PWIDs and prisoners) Reinfection risk in high-risk groups	Mobile clinics, harm-reduction services, and NGO partnerships increase access Continuous monitoring and reinfection prevention are crucial

## Data Availability

Data are contained within the article.

## Abbreviations

The following abbreviations are used in this manuscript:

HCV	Hepatitis C virus
WHO	World Health Organization
DAAs	Direct-acting antiviral agents
HCC	Hepatocellular carcinoma
PEG-IFNα	PEGylated interferon alpha
RBV	Ribavirin
HCV RNA	Hepatitis C virus ribonucleic acid
SVR12	Sustained virologic response at 12 weeks
SVR	Sustained virologic response
HBV	Hepatitis B virus
PWIDs	People who inject drugs
HIV	Human Immunodeficiency Virus
MSMs	Men who have sex with men
IDU	Injection drug use
ECDC	European Centre for Disease Prevention and Control
E1	HCV envelope glycoprotein 1
E2	HCV envelope glycoprotein 2
3′UTR	3′ untranslated region
5′UTR	5′ untranslated region
NAFLD	Non-alcoholic fatty liver disease
OAT	Opioid agonist therapy
LMICs	Low- and middle-income countries
ELISA	Enzyme-linked immunosorbent assay
CLIA	Chemiluminescence immunoassay
NAT	Nucleic acid amplification testing
HEOR	Health economics and outcomes research
USA	United States of America
UK	United Kingdom
TB	Tuberculosis
NGOs	Non-governmental organizations
DDT	Dry drop test
STIs	Sexually transmitted infections
BBV	Blood-borne Viral
EU	European Union
EHRs	Electronic health records
CDC	Centers for Disease Control and Prevention
mRNA	Messenger RNA
